# Diet Modification and Metformin Have a Beneficial Effect in a Fly Model of Obesity and Mucormycosis

**DOI:** 10.1371/journal.pone.0108635

**Published:** 2014-09-30

**Authors:** Fazal Shirazi, Dimitrios Farmakiotis, Yuanqing Yan, Nathaniel Albert, Do Kim-Anh, Dimitrios P. Kontoyiannis

**Affiliations:** 1 Department of Infectious Diseases, Infection Control and Employee Health, The University of Texas M.D. Anderson Cancer Center, Houston, Texas, United States of America; 2 Department of Biostatistics, The University of Texas M.D. Anderson Cancer Center, Houston, Texas, United States of America; University of Cordoba, Spain

## Abstract

In an experimental model of obesity and hyperglycemia in *Drosophila melanogaster* we studied the effect of diet modification and administration of metformin on systemic infection with *Rhizopus*, a common cause of mucormycosis in diabetic patients. Female *Wt*-type *Drosophila* flies were fed regular (RF) or high-fat diet (HFD; 30% coconut oil) food with or without metformin for 48 h and then injected with *R. oryzae*. Survival rates, glucose and triglyceride levels were compared between 1) normal-weight flies (RF), 2) obese flies (HFD), 3) obese flies fed with RF, 4) flies continuously on HFD + metformin, 5) flies fed on HFD + metformin, then transferred to RF, and 6) obese flies administered metformin after infection. Glucose levels were compared across groups of non-infected flies and across groups of infected flies. Survival was significantly decreased (*P* = 0.003) in obese flies, while post-infection glucose levels were significantly increased (*P* = 0.0001), compared to normal-weight flies. Diet and administration of metformin led to weight loss, normalized glucose levels during infection, and were associated with decreased mortality and tissue fungal burden. In conclusion, diet and metformin help control infection-associated hyperglycemia and improve survival in *Drosophila* flies with mucormycosis. Fly models of obesity bear intriguing similarities to the pathophysiology of insulin resistance and diabetes in humans, and can provide new insights into the pathogenesis and treatment of infections in obese and diabetic patients.

## Introduction

Infectious complications have always been among the leading causes of morbidity and mortality in diabetic patients [1]. Two thirds of the adult populations are overweight, and nearly 6% have diabetes [2]. Persistently high glucose levels suppress the activity of host immune cells against various pathogens [1], [3], [4] and create a favorable environment for fungal growth [5].

One of the most feared infections in poorly controlled diabetics is mucormycosis, which is caused by molds of the order Mucorales, most commonly *Rhizopus* spp. [5]–[7]. In one study, obesity and diabetes were present in two thirds of mucormycosis cases [3]. Mucormycosis also affects patients with a compromised immune system, primarily patients with hematologic malignancies and prolonged neutropenia, hematopoetic stem cell and solid organ transplant recipients, and patients on chronic steroids [6], [7]. Of note, hyperglycemia has been identified as an independent risk factor for mucormycosis in those high-risk groups [5], [8]. Mortality due to mucormycosis can be as high as 90% [8], [9], and the cornerstone of disease management in severe disease is amphotericin-B [7], which is associated with renal toxicity and infusion reactions [9]. Therefore, further elucidation of pathophysiologic mechanisms, and development of novel prevention and treatment strategies are urgently needed to improve outcomes in patients with this devastating infection.

Our group has previously described a novel mucormycosis model, in which *Drosophila melanogaster* is infected with Mucorales spores, in order to study virulence and host defense mechanisms [10]. We found that classic enhancers of pathogenicity in humans, such as corticosteroids and increased iron supply, dramatically increased mortality, and we established *D. melanogaster* as a relevant, high throughput and low-cost model to study the pathogenesis of mucormycosis [10].

*D. melanogaster* also serves as an elegant system for the study of obesity, which is the most important modifiable risk factor for development of type 2 diabetes (DM2) in humans [11]–[14]. Obesity seems to be independently associated with impaired immune responses to infection [15]. Given the paucity of data regarding the interplay between obesity, virulence factors and host defense mechanisms against the Mucorales, we developed a model of disseminated *Rhizopus oryzae* infection in obese and hyperglycemic *Drosophila* flies. We hypothesized that obesity, with or without hyperglycemia, is associated with increased mortality from mucormycosis, while induction of weight loss with diet modification or administration of metformin can improve survival rates.

## Materials and Methods

### D. melanogaster Stocks

Oregon^R^ wild-type *D. melanogaster* flies were used in all experiments. Standard procedures for manipulation, feeding, and housing of the flies were used in all experiments [10].

### Isolates and Growth Conditions

A clinical isolate of *R. oryzae* (isolate 969) was grown on freshly prepared Sabouraud dextrose agar plates. After 48 h of incubation at 37°C, spores were collected and washed twice in sterile phosphate-buffered saline. Subsequently, spores were counted using a hemocytometer and stored at 4°C in phosphate-buffered saline.

### Metformin Treatment

Metformin (Sigma) was added directly to regular food from a 1 M aqueous stock to a final concentration of 1, 5, 10, 25, 50 and 100 mM. For control food, we used water alone.

### Toxicity Assays

For toxicity experiments, flies were maintained in vials containing 30 flies per vial on standard regular food (RF) and high fat diet (HFD) media, containing different concentrations of metformin. Flies were transferred to new vials three times per week, and the number of dead flies was determined on a daily basis.

### High-Fat Diet Feeding Regimen

**Figure 1:**
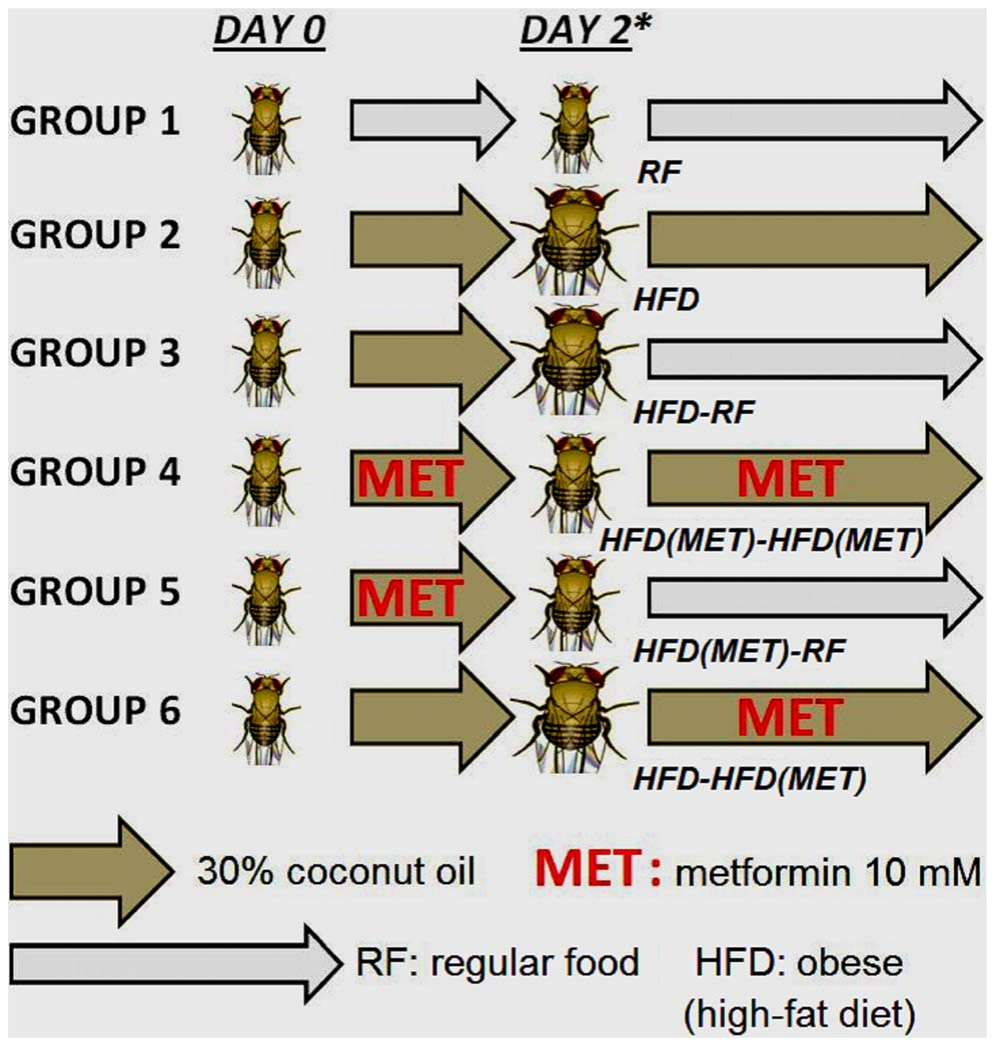
Experimental design. The following six treatment groups were studied: group 1, RF (control, normal-weight flies); group 2, HFD; group 3, HFD followed by a switch on day 2 to RF (HFD-RF); group 4, HFD-continuous metformin (HFD(MET)-HFD(MET); group 5, HFD-metformin followed by a switch on day 2 to RF (HFD(MET)-RF); and group 6, HFD followed by a switch on day 2 to HFD-metformin (HFD-HFD(MET). *In a separate experiment, flies were also injected with *Rhizopus* spores on day 6, when the flies were highly hyperglycemic.

Vials were emptied and dated; 5 days after the vials were emptied, all newborn flies were collected and placed in a new vial containing RF for 2 more days. This [fly] population was divided into three subgroups: one was fed with RF; the other two were fed with HFD and HFD (MET) for two days and were transferred to individual treatment groups for 8 days. We used 30% coconut oil as HFD for all experiments, since this feeding regimen has been shown to result in highly reproducible phenotypes [16]. Survival was compared across six groups (Fig. 1) (30 flies each): RF, HFD, HFD followed by a switch on day 2 to RF, HFD-metformin, HFD-metformin followed by a switch on day 2 to RF, and HFD followed by a switch on day 2 to HFD-metformin.

### Infection Model

Female wild-type *D. melanogaster* flies were starved for 6–8 h and were fed RF, HFD with and without MET, then infected with *R. oryzae* spores after 48 h on their feeding regimen by injection in the thorax with a 10 µm needle previously dipped in a solution containing 10^4^–10^7^ spores/ml, as previously described [10]. In previous experiments, quantitation of Mucorales spores in the infection inocula revealed that at such spore concentrations in the solution, flies were inoculated with ∼5×10^2^ to 8×10^2^ spores [10]. Flies that died within 3 h of infection (<5%) were excluded from survival analysis. After infection, the flies were transferred to fresh fly food vials. Survival was assessed daily until day 4 after infection. All experiments were performed in triplicate on three different days. To minimize circadian rhythm variability, all experiments were performed at 3:00 p.m.

### Glucose-Trehalose Measurements

For glucose measurements [16], 20 wild-type female flies were homogenized in 100 µl of 100 mM PIPES buffer (Sigma) with porcine kidney trehalase at 5 µl per 2 ml (Sigma) for 1 min at 10,000 rpm using a glass bead homogenizer. Trehalase converts trehalose (present in the hemolymph) into glucose, and thus total available glucose levels are measured. The homogenates were incubated in a 37°C water bath for 1 h, and 10 µl were transferred into 90 µl of Glucose Oxidase Reagent (Pointe Scientific, Inc.). The reaction mixture was incubated at 37°C for 5 min; absorbance was measured at 500 nm using a KC4 plate reader (Bio-Tek Instruments, Inc.).

### Triglyceride Measurements

Treated adult flies were placed into empty vials for 30 min and then placed into eppendorf tubes in batches of 12 and weighed for immediate quantification or frozen at −80°C for later processing, as previously described [16]. Flies were then homogenized in 100 µl of a 1∶1 (vol:vol) mixture of chloroform/methanol for 1 min at 10,000 rpm using a glass bead homogenizer. The homogenates were subsequently centrifuged at 4000 rpm in an Eppendorf 5810 R centrifuge for 5 min, and 5 µl of the supernatant was transferred into 170 µl of Triglycerides Reagent (Thermo Electron Corp.). The reaction mixture was incubated at 37°C for 10 min; optical density at 550 nm was measured using a KC4 plate reader (Bio-Tek Instruments, Inc.).

### Histopathological Analysis

On day 3 after infection with Mucorales spores, samples were fixed in 10% formaldehyde, processed, and embedded in paraffin. Tissue sections were stained with hematoxylin-eosin and Grocott-Gomori methenamine-silver nitrate and examined for visible fungal burden under a light microscope.

### Statistical Analyses

For all assays, three independent experiments were carried out on three different days. All data plots were created with SAS version 9.3 (SAS Institute, Cary, NC). All statistical analyses were carried out with SAS software. ANOVA was used to analyze one-time sampling data. A linear mixed-effects model was used for time course sampling data. Survival data were analyzed by the log-rank test. Survival curves based on estimated survival rates and 95% confidence intervals (log S(t)) were plotted. For all comparisons, *P* values of <0.05 were considered statistically significant.

## Results

### Flies Become Obese after Two Days on a High-Fat Diet and Subsequently Develop Hyperglycemia

**Figure 2:**
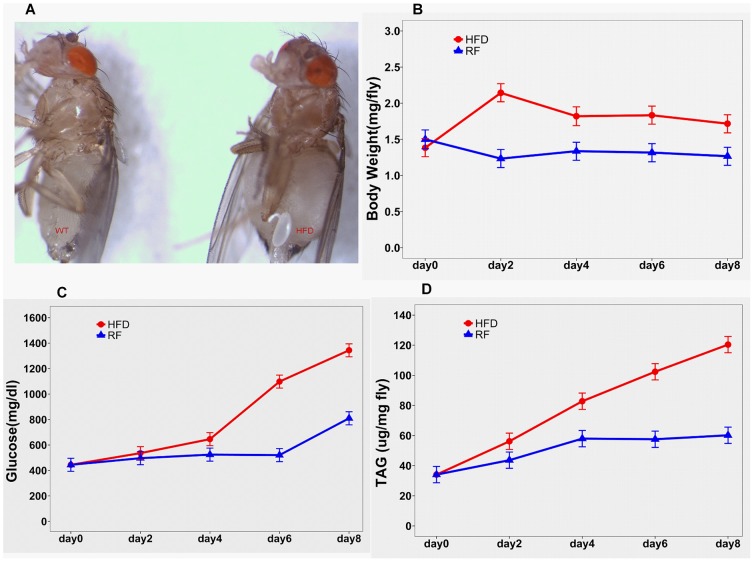
Effects of HFD on the flies' body weight and glucose and triglyceride levels (means and 95% confidence intervals were calculated using either general linear or mixed linear models). A) Images of WT and HFD flies; B) body weight over time; C) glucose levels over time; D) triglyceride (TAG) levels over time. *P<0.05 compared to obese (HFD) flies.

Flies fed a high-fat diet (HFD) for 2 days gained about 0.65±0.12 mg of weight and became obese, compared to flies fed on RF (*P* = 0.0001; Fig. 1, 2A, and 2B). The mean body weight of flies fed HFD was higher than the weight of RF flies for a long period (Fig. 2B). In the HFD group, the mean body weight on day 0 was lower than the average of mean body weights for subsequent days by 0.50±0.07 mg (*P*<0.0001). The pairwise differences in mean body weight between the HFD and RF groups were all significant (*P*<0.0001), with the greatest difference on day 2 (Fig. 2A).

We used the glucose oxidase-trehalase assay to measure total glucose levels. Trehalase converts trehalose (a D-glucose disaccharide present in the endolymph) into glucose, and total available glucose levels are measured with the glucose oxidase reagent [16]. Differing glucose levels reported in the literature can easily be accounted for by multiple factors, including the developmental stage and age of the flies, as well as the method of extraction [16], [17]. Our results showed that glucose levels progressively increased in both HFD- and RF-fed flies (Fig. 2C). There was no significant difference in glucose levels between the HFD and RF groups until day 2 (*P* = 0.43; Fig. 2C). Starting on day 4, the differences in glucose levels between the HFD and RF groups were all significant, peaking on day 6 (difference of 577.6±47.1 mg/dl) (*P*<0.0001). Thus, HFD flies were considered significantly hyperglycemic on day 6.

In *Drosophila*, lipids are primarily stored as triglycerides (TAGs) in the fat body [16]. TAG levels also progressively increased in flies fed on HFD (Fig. 2D). Starting on day 2, TAG levels increased significantly more in HFD flies, than in RF flies (P<0.0001) (Fig. 2D). Our findings are in agreement with those previously reported by Birse et al [16].

### Metformin Toxicity Studies

In agreement with a previous report [18], we did not observe a long-term survival benefit with administration of metformin at a concentration of 5–10 mM in the feeding media, and higher doses seemed to be associated with late toxicity (Fig. S1, S2). Metformin decreased circulating triglyceride levels in both HFD and RF flies, in a dose-dependent manner (Fig. S1). For our subsequent infection experiments, we used a metformin concentration of 10 mM, which decreased triglyceride levels significantly without leading to excess long-term mortality (Fig. S1, S2).

### Diet Modification and Metformin Normalize Body Weight and Glucose and Triglyceride Levels

To explore whether the HFD-induced weight gain and increases in glucose and triglyceride levels could be reversed, we investigated the effects of diet modification and metformin administration in four treatment groups: HFD with a switch to RF on day 2 (HFD-RF), continuous HFD-metformin (HFD (MET)-HFD (MET)), HFD-metformin with a switch to RF (no metformin) on day 2 (HFD(MET)-RF), and HFD with a switch to HFD-metformin on day 2 (HFD-HFD (MET)) (Fig. 1).

**Figure 3:**
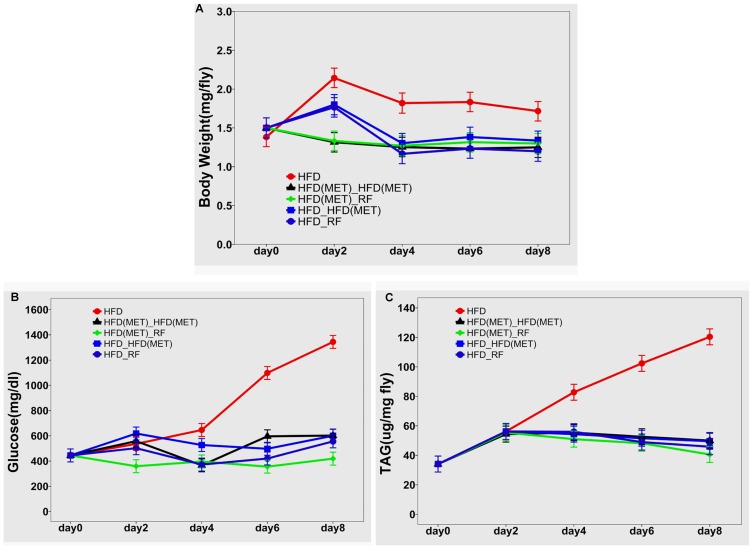
Effects of diet modification and metformin administration on body weight and glucose and triglyceride levels (means and 95% confidence intervals were calculated using either general linear or mixed linear models). A) mean body weights over time; B) mean glucose levels over time; C) mean triglyceride (TAG) levels over time. *P<0.05 compared to all other groups. In Panel A, the line representing the HFD (MET)-HFD (MET) group overlaps with the line corresponding to the HFD-RF group.

For one-time sampling data, the Tukey's Studentized range test at a type I error level of 0.05 showed that the mean body weights in all four treatment groups were equivalent to that in the RF control group but significantly lower than that in the HFD control group (Fig. 3A).

The next experiments were designed to explore the effects of diet modification and metformin administration on the HFD-induced increases in body weight and glucose and triglyceride levels over time. In the HFD (MET)-RF and HFD (MET)-HFD (MET) groups (which received metformin starting on day 0), mean body weights were 0.92±0.12 mg lower than in the HFD-RF and HFD-HFD (MET) groups (*P*<0.0001; Fig. 3A). The difference in body weight between the HFD-RF and HFD-HFD (MET) groups (which did not receive metformin during the first 2 days) was not significant (0.03±0.09 mg; *P* = 0.71). Similarly, the difference in body weight between the HFD (MET)-RF and HFD (MET)-HFD (MET) groups (both of which received metformin on the first 2 days) were not significant (0.02±0.09 mg; *P* = 0.85). These findings suggested that body weight remained stable over time in both the HFD (MET)-RF group and the HFD (MET)-HFD (MET) group.

In the HFD-RF and HFD-HFD (MET) groups, body weight normalized by day 4, similarly to the HFD (MET)-RF and HFD (MET)-HFD (MET) groups (*P*>0.67) (Fig. 3A). Glucose levels in all four intervention groups were significantly lower than that in the reference HFD group. There was a trend for higher glucose levels in the groups of flies that were administered metformin, compared to those transferred to regular food on days 6 and 8, which could be attributed to the different glucose content between 30% coconut oil and regular food (Fig. 3B).

Lipid stores are decreased in flies raised in the presence of metformin. In our study, after 6 days of treatment, triglyceride levels in both RF (normal-weight) and HFD (obese) female flies given 10 mM or 100 mM metformin were significantly lower than in untreated controls (Fig. S1). On day 2, mean triglyceride levels in all four treatment groups did not differ from each other, but starting on day 4 they were all significantly lower than that in the HFD group (Fig. 3C). These findings indicated that metformin has dose-dependent effects on fat metabolism in *Drosophila*.

### Diet Modification and Metformin Prevent Acute Hyperglycemia in Obese *Drosophila* Flies Infected with *Rhizopus*

**Figure 4:**
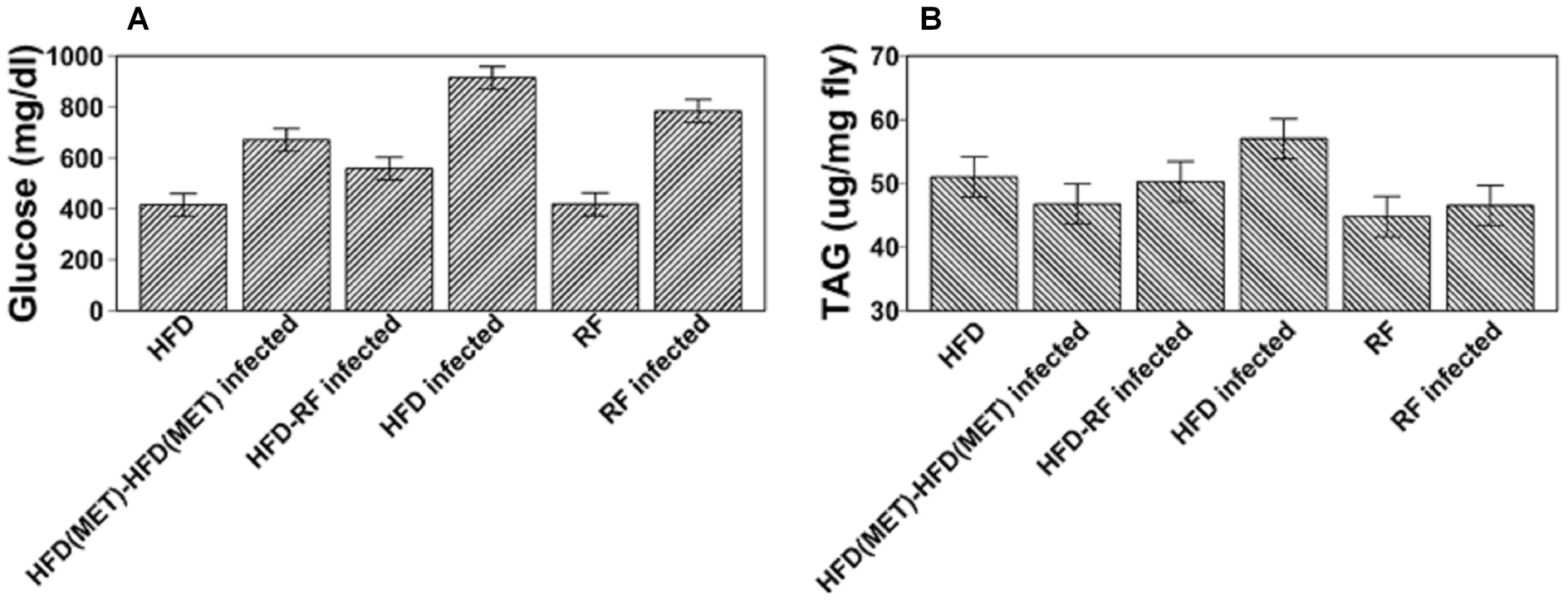
Glucose (A) and triglyceride (B) levels in uninfected control and *R. oryzae* infected flies ((means and 95% confidence intervals were calculated using general linear models), 24 hours after injection. *P<0.05 compared to non-infected flies, ^¶^P<0.05 compared to obese (HFD) infected flies.

In both the RF group and the HFD group, infection with *R. oryzae* led to a significant increase in glucose at 24 h compared to the glucose level in uninfected flies (*P* = 0.0001; Fig. 4A), similar to the well-described stress-induced hyperglycemia in humans [19], [20]. Also, a similar hyperglycemic response of obese flies to different kinds of stress has been previously reported [17]. The glucose level in infected flies on HFD was significantly higher than that in infected flies fed with RF (*P* = 0.0005). A two-way analysis of variance (ANOVA) showed that there was a significant association between infection and obesity in influencing glucose levels (*P*<0.0001). Importantly, compared to the mean glucose level (913 mg/dl) in HFD-fed flies 24 h after *R. oryzae* infection, the mean glucose levels in the HFD-RF and HFD(MET)-HFD(MET) flies infected with *R. oryzae* were significantly lower (557.8 mg/dl and 670.2 mg/dl, respectively; *P*<0.0001). At 24 h after *R. oryzae* infection, triglycerides in RF flies were approximately 46 µg/mg (*P* = 0.42; Fig. 4B). Continuous administration of metformin (HFD (MET)-HFD (MET)) and diet modification (HFD-RF) were associated with significantly lower triglyceride levels (46.75 and 50.25 µg/mg, respectively) than HFD (57 µg/mg; *P* = 0.0001 and *P* = 0.0053, respectively) 24 h after infection (Fig. 4B).

### Metformin and Diet Modification Are Associated with Increased Survival and Decreased Fungal Burden in Experimental Mucormycosis

An injection assay was performed on RF- and HFD-fed flies (normal-weight and obese flies, respectively) with 5×10^7^, 5×10^6^, 5×10^5^, and 5×10^4^ *R. oryzae* spores/ml. Results showed inoculum-dependent survival in both normal-weight and obese flies; however, obese flies died faster than normal-weight flies (Fig. S3). All subsequent experiments were performed with an inoculum of 5×10^7^ spores/ml.

**Figure 5:**
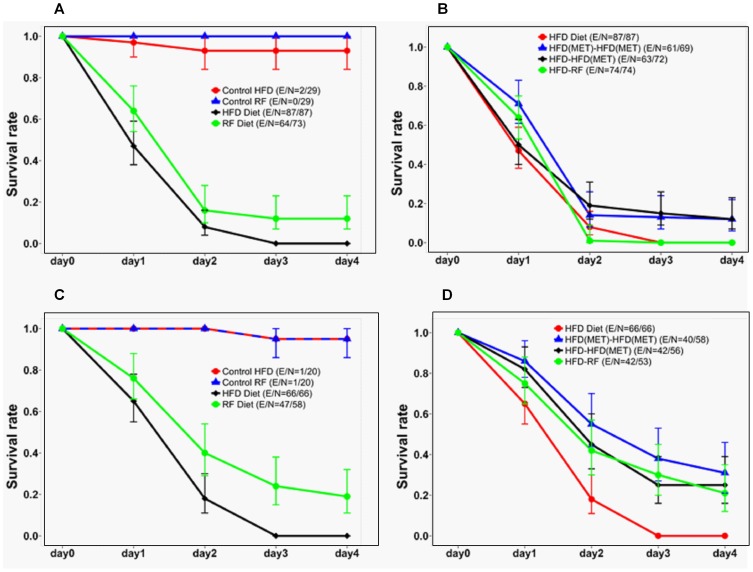
Beneficial effects of metformin administration and diet modification on survival of flies infected with *R. oryzae*. Flies in the control HFD and control RF groups were not infected. The data are mean estimated survival rates with 95% confidence intervals. The E/N ratio indicates the total number of deaths/total number of flies. A) Survival rates of uninfected, obese non-hyperglycemic (HFD)- or RF-fed flies (Control HFD and Control RF, respectively), compared with *Rhizopus*-infected obese non-hyperglycemic (HFD)- or RF-fed flies (High-Fat Diet and RF Diet, respectively); B) effects of metformin administration and diet modification on the survival rates of *Rhizopus*-infected obese non-hyperglycemic flies; C) survival rates of uninfected, obese hyperglycemic HFD- or RF-fed flies compared with *Rhizopus*-infected, obese hyperglycemic HFD- or RF-fed flies; D) effects of metformin administration and diet modification on the survival rates of *Rhizopus*-infected obese hyperglycemic flies.

In the absence of fungal infection, there was no difference in survival rates between obese (HFD) and non-obese (RF) flies (*P* = 0.61; Fig. 5A). For both groups, infection was a significant risk factor for decreased survival (*P*<0.0001). Furthermore, infection decreased the survival rate of obese flies more than non-obese flies (*P* = 0.003). To test the effects of diet modification and metformin in mitigating the combined risk of obesity and fungal infection, obese infected flies received the following interventions: HFD-RF, HFD (MET)-HFD (MET), and HFD-HFD (MET). While the HFD-RF treatment did not improve survival in obese infected flies (*P* = 0.14, Bonferroni adjusted *P* = 0.85), the HFD-HFD (MET) group showed improved survival (*P* = 0.03 or Bonferroni adjusted *P* = 0.16), similar to the HFD (MET)-HFD (MET) group (*P* = 0.001, Bonferroni adjusted *P* = 0.006; Fig. 5B).

We also examined the effects of diet modification and metformin administration on survival of HFD-fed flies that became significantly hyperglycemic and then were infected with *Rhizopus* (Fig. 5C and 5D). With no fungal infection, there was no difference in survival between the HFD and RF flies (*P* = 1.00; Fig. 5C). Fungal infection significantly decreased the survival rate in obese hyperglycemic flies, compared to RF (*P* = 0.0012, Bonferroni adjusted *P* = 0.0072; Fig. 5C). In the HFD(MET)-HFD(MET), HFD-HFD(MET), and HFD-RF groups, survival rates were significantly higher than in the HFD group (the corresponding P values were as follows: *P*<0.0001, Bonferroni adjusted *P*<0.0001; *P*<0.0001, Bonferroni adjusted *P* = 0.0003; and *P* = 0.0003, Bonferroni adjusted *P* = 0.0018) (Fig. 5D).

**Figure 6:**
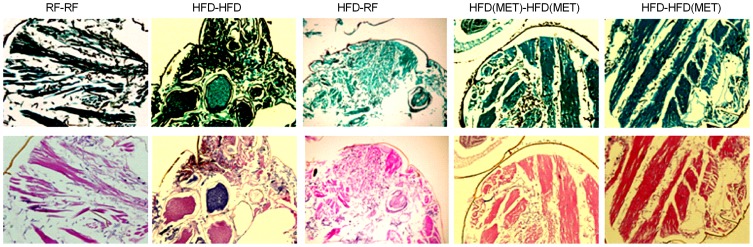
Representative hematoxylin-eosin (upper panels)- and Grocott-Gomori methenamine-silver nitrate (lower panels)-stained cross-sections of *Drosophila* flies infected with *R. oryzae*. Flies fed RF or given metformin, particularly those continuously kept on metformin (HFD(MET)-HFD(MET)) had a lower fungal burden than infected flies fed HFD without metformin.

Hematoxylin-eosin and Grocott-Gomori methenamine-silver nitrate staining demonstrated that infected flies that received RF continuously, infected flies whose diets were switched from HFD to RF, and infected flies that received metformin with HFD, particularly those that received metformin continuously (HFD(MET)-HFD(MET)), had lower fungal burdens than infected flies that were fed HFD continuously without metformin (Fig. 6).

## Discussion

According to the World Health Organization, one of every eight adults worldwide is obese [21]. This global “epidemic” of obesity is associated with significant morbidity and functional decline and is the most important risk factor for the development of DM2 [22], [23]. At its early stages, obesity results in resistance of peripheral tissues to insulin, through an increase in circulating triglycerides [23], and the release of adipokines, peptides with a multitude of endocrine and paracrine effects [24]. Once insulin secretion by the pancreatic islet β-cells can not compensate adequately for the degree of insulin resistance, hyperglycemia and DM2 are established [25].

Animal systems that model obesity can contribute significantly to the investigation of complex relationships between increased adiposity, insulin resistance and long-term complications from diabetes [18]–[21]. In the present study, we adapted our previously described [10] fly model of mucormycosis to the fly model of HFD-induced obesity developed by Birse et al [16]. We demonstrated markedly increased susceptibility of obese and hyperglycemic flies to this rapidly progressing infection, as well as a protective effect of weight loss induced by diet modification and/or administration of metformin (Fig. 2, 3, 4, 5).

Similarly to humans, flies store and mobilize energy in the form of carbohydrates and lipids, and utilize highly conserved insulin and glucagon-like signaling pathways to regulate their glucose homeostasis [11]–[14]. Therefore, *Drosophila melanogaster* has emerged as an attractive model for the study of obesity and hyperglycemic states [11]–[17]. In the present study, we showed that *Drosophila* flies fed on a HFD became obese after 48 h, and developed significant hyperglycemia four days later (Figs. 2, 3). Our results are in agreement with those reported by the group that first developed the HFD-induced obesity fly model [16], and simulate the well-known progression of obesity and insulin resistance to diabetes in humans [22], [15].

We subsequently tested the effect of the insulin-sensitizing agent metformin, a widely prescribed medication that is considered first-line treatment for DM2 [26], on our fly model of obesity and disseminated mucormycosis. Metformin administration induces physiological phenotypes similar to those produced by diet restriction [18], [26]; specifically, metformin has been shown to lower blood glucose (predominantly by decreasing glucose production by the liver), increase insulin-dependent glucose uptake in peripheral tissues, lower circulating insulin levels, and promote fatty acid metabolism [18]. In our study, we confirmed that metformin has physiologic effects very similar to those of dietary restriction, leading to weight loss (Figs. 2, 3), as well as normalization of total glucose (Fig. 3) and triglyceride levels (Fig. S1). In agreement with a previous study in rhesus monkeys [27], long-term mortality was higher in obese than normal-weight flies. However, in contrast to previously reported findings in rodents [28], [29], we did not observe a survival benefit with metformin. In fact, higher doses of metformin were associated with toxicity, in agreement with previous experiments in flies [18] (Fig. S2). Importantly, administration of metformin was associated with decreased mortality in acute *R. oryzae* infection, both in obese, non-hyperglycemic flies (infected on day 2, Fig. 5A, B) and after development of significant hyperglycemia on day 6 (Fig. 5C, D).

An important finding from our study was that obese *Drosophila* flies had significantly higher glucose levels 24 h after injection with *Rhizopus*, than in the absence of infection (Fig. 4). This type of infection-induced hyperglycemia is a well-described feature of “pre-diabetes” in humans [19] and has been previously reported in the same phenotype of diet-induced obesity, in response to cold or hypoxia, both of which were associated with increased mortality in obese flies, compared with normal-weight flies [17]. In our model, dietary modification and metformin helped control total glucose levels in acute infection (Fig 4) and led to improved survival rates (Fig. 5), as well as decreased fungal burden (Fig. 6). Therefore, hyperglycemia seems to be a ubiquitous response to life-threatening stress, and likely is one of the factors leading to increased mortality in our fly model of obesity and disseminated mucormycosis.

In DM2, persistently elevated glucose levels have been shown to suppress both adaptive and innate immunity [1]. Specifically, reduced cytokine production, as well as impaired polymorphonuclear chemotaxis, tissue transmigration and superoxide production have all been described in diabetic patients with poor metabolic control [1], [4]. Moreover, our group previously showed that *Drosophila* S2 cells display decreased phagocytosis and cause less hyphal damage to *R. oryzae*, compared with that to the non-pathogenic *Aspergillus fumigatus* [10]. Taken together, those results indicate that hyperglycemia-induced phagocyte dysfunction might play a central role in the increased susceptibility to Mucorales infection observed in our obese and diabetic flies.

In clinical practice, obesity has recently been identified as an independent risk factor for increased incidence of infections and worse outcomes of various nosocomial and community-acquired infections [15]. Obese individuals have a significantly higher risk for respiratory [30 31], periodontal [32], postoperative wound [33], and urinary tract [34] infections. Those associations are definitely influenced by mechanical complications and management pitfalls that are more frequently encountered in the obese, such as mobility problems, imaging difficulties, and prolonged duration of the presence of central lines and indwelling catheters. However, there is also evidence linking excess adiposity with immune system dysfunction, specifically phagocytosis, cytokine production and T-cell number and activation [15]. For example, obese rodents have been shown to be more susceptible to bacterial [35] and *Candida* [36] infections.

Hyperglycemia in sepsis is considered by some an evolutionary conserved defense mechanism, for securing an adequate glucose supply to hypo-perfused and injured tissue [20]. Additional mechanisms, possibly related to insulin resistance and hyperinsulinemia, may contribute to immune dysfunction and the increased susceptibility to infection in obesity. Of interest, the effect of insulin signaling in insects appears to vary with the pathogen tested. For example, activation of *Drosophila* insulin-like peptide (DILP) signaling pathways has been shown to protect flies from *Mycobacterium marinum* infection and the associated hyperglycemic wasting syndrome [37]. In contrast, increased insulin-like activity seems to be detrimental in *Staphylococcus aureus* or *Enterococcus faecalis* systemic infection in flies [38]. Little is known about the potential role of hyperinsulinemia in mucormycosis. Therefore, in our study, one cannot exclude a significant contribution to increased mortality by obesity-related factors other than hyperglycemia, such as insulin resistance and enhanced DILP activity. It is possible that, besides preventing stress-related hyperglycemia, dietary modification and metformin exert some of their beneficial effects against mucormycosis by suppressing DILP pathways. Furthermore, it is possible that suppression of insulin resistance-related mechanisms could also account for the observed survival benefit of pharmacologic insulin sensitization with metformin prior to infection, compared with the effect of diet modification alone (Fig. 5), even though there was a trend for higher glucose levels in the groups of flies that were administered metformin, compared to those transferred to regular food (Fig. 3B).

In summary, we have described for the first time increased susceptibility to an invasive mold infection in an animal model of obesity. We have shown that induction of weight loss by modification of diet or administration of metformin decreased mortality in obese and hyperglycemic *Drosophila* flies after disseminated infection with *R. oryzae*. Administration of metformin appears to be more beneficial than diet modification alone. Additional studies with the *Drosophila* obesity phenotype could help us determine more precisely the connection between proinflammatory or immunosuppressive states in obesity and the risk of infection. Fly models of obesity bear intriguing similarities to the pathophysiology of insulin resistance and DM2 in humans and could provide new insights into the pathogenesis and treatment of infections in obese and diabetic patients.

## Supporting Information

Figure S1 **Effects of different metformin concentrations in feeding media (wt/vol) on circulating triglyceride (TG) levels in obese (HFD) and normal-weight (RF) flies.** ***P<0.0001 compared to no metformin within the same group (RF or HFD); ^¶^ *P*<0.05 compared to RF.
(TIF)

Figure S2 **Metformin toxicity (as evidenced by the percentage of flies surviving) in normal-weight (A) and obese (B) flies (the indicated concentrations in feeding media are wt/vol).** *P<0.05, **P<0.001, and ***P<0.0001, respectively, compared to controls.
(TIF)

Figure S3 **Effects of different inocula of** ***R. oryzae*** **on survival of** ***Drosophila*** **flies.**
(TIF)
