# Correction: Diet Modification and Metformin Have a Beneficial Effect in a Fly Model of Obesity and Mucormycosis

**DOI:** 10.1371/journal.pone.0116684

**Published:** 2014-12-31

**Authors:** The PLOS ONE Staff

The fifth author’s name is spelled incorrectly. The correct name is: Kim-Anh Do. The correct citation is: Shirazi F, Farmakiotis D, Yan Y, Albert N, Do KA, et al. (2014) Diet Modification and Metformin Have a Beneficial Effect in a Fly Model of Obesity and Mucormycosis. PLoS ONE 9(9): e108635. doi:10.1371/journal.pone.0108635

There are errors in the legend for Figure 6, “Representative hematoxylin-eosin (upper panels)- and Grocott-Gomori methenamine-silver nitrate (lower panels)-stained cross-sections of *Drosophila* flies infected with *R. oryzae*.” The complete, correct Figure 6 legend is: “Flies fed RF or given metformin, particularly those continuously kept on metformin (HFD(MET)-HFD(MET)) had a lower fungal burden than infected flies fed HFD without metformin.”

There is an error in the third sentence of the Abstract. The correct sentence is: “Survival rates, glucose and triglyceride levels were compared between 1) normal-weight flies (RF), 2) obese flies (HFD), 3) obese flies fed with RF, 4) flies continuously fed on HFD + metformin, 5) flies fed on HFD + metformin, then transferred to RF, and 6) obese flies administered metformin after infection.”
