# Correction: Diet Modification and Metformin Have a Beneficial Effect in a Fly Model of Obesity and Mucormycosis

**DOI:** 10.1371/journal.pone.0317530

**Published:** 2025-01-09

**Authors:** Fazal Shirazi, Dimitrios Farmakiotis, Yuanqing Yan, Nathaniel Albert, Do Kim-Anh, Dimitrios P. Kontoyiannis

Contrary to this article’s Data Availability statement [1], the individual-level data underlying the published results were not provided with the article or its subsequent correction [1, 2]. This notice is issued to share the individual-level data underlying the results presented in Figs 2–5 (S1 File), bringing the article into compliance with the PLOS Data Availability policy in place at the time of the article’s submission.

The corresponding author stated that the individual-level data underlying Figures S1-S3 of [1] are no longer available due to the time that has elapsed since these experiments were conducted.

## Supporting information

S1 File Individual-level data underlying Figs 2–5 presented in [1]. (XLSX)
