# Supplementary figures and images for: Diet Modification and Metformin Have a Beneficial Effect in a Fly Model of Obesity and Mucormycosis
Source: PLoS One. 2014 Sep 30;9(9):e108635. doi: 10.1371/journal.pone.0108635 (PMC4182538; doi:10.1371/journal.pone.0108635)

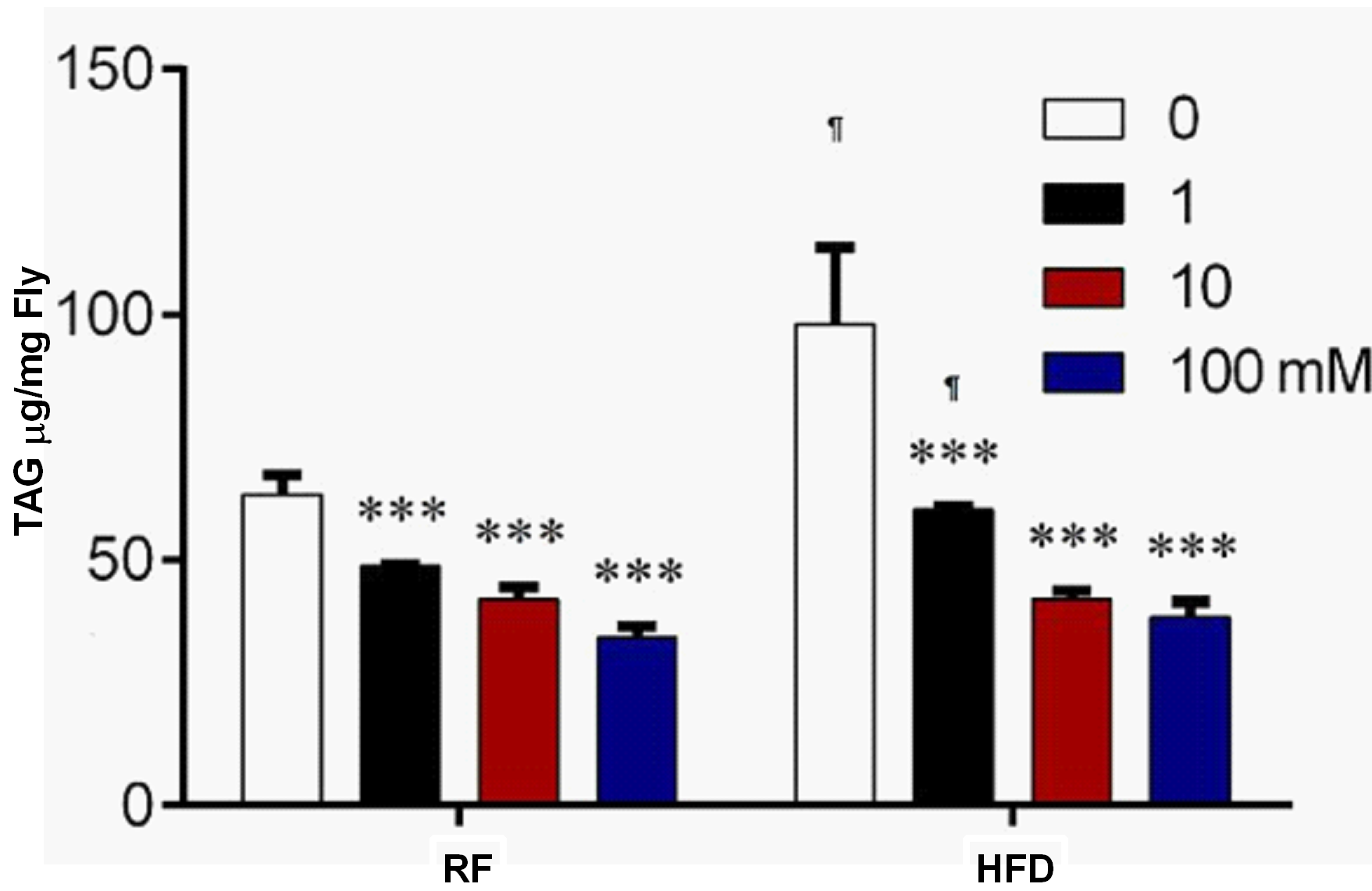

Supplement: Figure S1 — Effects of different metformin concentrations in feeding media (wt/vol) on circulating triglyceride (TG) levels in obese (HFD) and normal-weight (RF) flies. ***P<0.0001 compared to no metformin within the same group (RF or HFD); ¶ P<0.05 compared to RF. (TIF) [file pone.0108635.s001.tif]

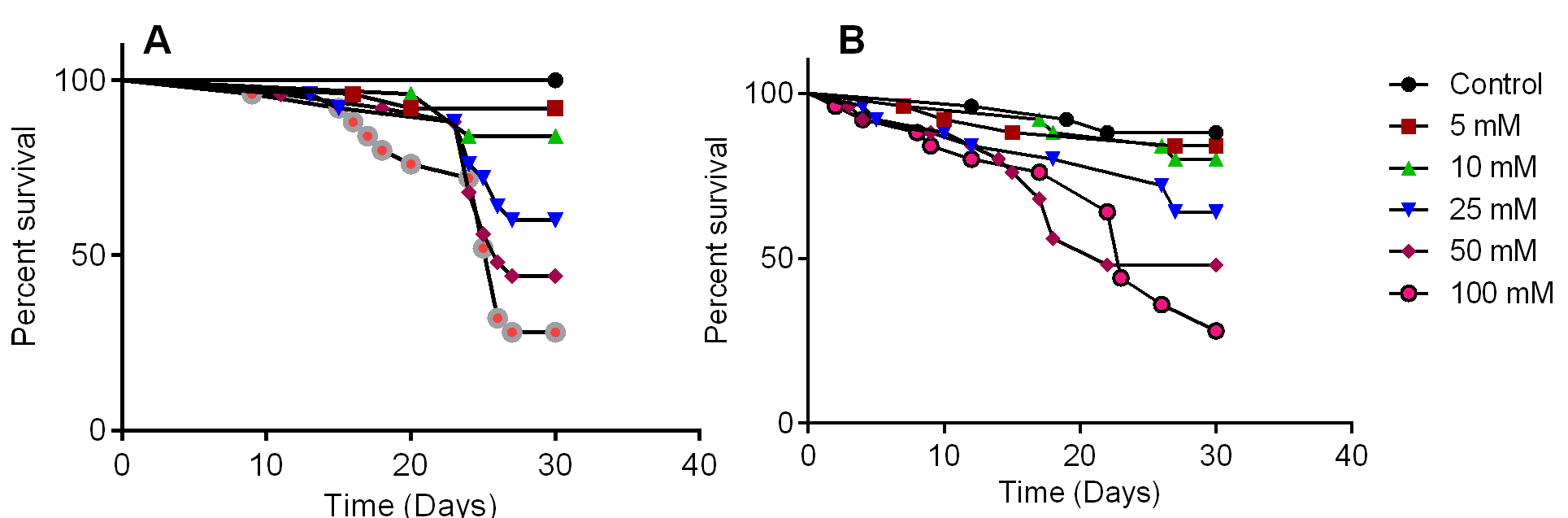

Supplement: Figure S2 — Metformin toxicity (as evidenced by the percentage of flies surviving) in normal-weight (A) and obese (B) flies (the indicated concentrations in feeding media are wt/vol). *P<0.05, **P<0.001, and ***P<0.0001, respectively, compared to controls. (TIF) [file pone.0108635.s002.tif]
